# Non-contiguous finished genome sequence and description of *Clostridiumihumii* sp. nov.

**DOI:** 10.1186/s40793-015-0025-x

**Published:** 2015-09-19

**Authors:** Vicky Merhej, Anne Pfleiderer, Dhamodharan Ramasamy, Jean-Christophe Lagier, Caroline Michelle, Didier Raoult, Pierre-Edouard Fournier

**Affiliations:** Unité de Recherche sur les Maladies Infectieuses et Tropicales Emergentes (URMITE), UM 63, CNRS 7278, IRD 198, Inserm 1095, Institut Hospitalo-Universitaire Méditerranée-Infection, Faculté de médecine, Aix-Marseille Université, 27 Boulevard Jean Moulin, 13385, Marseille, cedex 05 France

**Keywords:** *Clostridium ihumii*, Genome, Culturomics, Taxono-genomics

## Abstract

*Clostridium ihumii* strain AP5^T^ sp. nov. is a new species within the genus *Clostridium*. This strain, whose genome is described here, was isolated from the stool sample of a 21-year-old French Caucasian female with anorexia nervosa. *C*. ihumii is a Gram-positive, anaerobic bacillus. Here we describe the features of this organism, together with the complete genome sequence and annotation. The 4,433,668 bp long genome contains 4,076 protein-coding and 85 RNA genes, including 9 rRNA genes.

**Electronic supplementary material:**

The online version of this article (doi:10.1186/s40793-015-0025-x) contains supplementary material, which is available to authorized users.

## Introduction

*Clostridium**ihumii* strain AP5^T^ (=CSUR P198 = DSM 26098) is the type strain of *C. ihumii* sp. nov. This bacterium is a Gram-positive, anaerobic rod-shaped bacteria that was isolated from the stool sample of 21-year-old French Caucasian female with anorexia nervosa since the age of 12 years (body mass index 10.4 kg/m2), as a part of a “culturomics” study aiming at cultivating all species within human feces individually [1–3]. Using a large scale of culture conditions, MALDI-TOF MS and 16S ribosomal RNA (rRNA) sequencing, *C.* ihumii and 10 new bacterial species have been successfully identified in a single stool sample [4, 5].

Bacterial taxonomy has long relied on phenotypic, genotypic approaches such as DNA base composition (mol% G + C content), DNA-DNA hybridization, and the 16S rRNA gene-sequence identity [6–9]. The advent of high-throughput sequencing techniques has delivered new taxonomic metrics such as average nucleotide identity (ANI); thus a new method (taxono-genomics) based on a combination of genomic and phenotypic properties has proven to be useful for the description of new bacterial species [10–14].

Since the creation of the genus *Clostridium* in 1880, more than 200 species have been described [15]. Species belonging to this genus are obligate anaerobic, Gram-positive, rod-shaped, spore-forming bacteria. They are associated to the commensal digestive flora of mammals and can be commonly found in the environment. However, *C. botulinum*, *C. difficile* and *C. tetani* are causative agents of serious infectious diseases [16, 17].

Here we present a summary classification and a set of features for *C.* ihumii sp. nov. strain AP5^T^ together with the description of the complete genome sequence and annotation. These characteristics support the circumscription of the species C. *ihumii*.

## Organism information

### Classification and Features

A stool sample was collected from a 21-year-old French Caucasoid female who suffers from severe restrictive form of anorexia nervosa since the age of 12 years. At the time of sample collection, she was hospitalized in our hospital for recent aggravation of her medical condition (BMI: 10.4 kg/m^2^). The patient gave an informed consent. The study and the assent procedure received the agreement of the local ethics committee of the IFR48 (agreement number 09–022, Marseille, France). The stool sample of this patient was stored at −80 °C immediately after collection and studied by microbial culturomics, as previously reported. Strain AP5^T^ was isolated in January 2012 by anaerobic cultivation on 5 % sheep blood-enriched Columbia agar (BioMerieux, Marcy l’Etoile, France) after one month preincubation of the sample in blood culture bottle enriched with rumen fluid (Becton Dickinson, Temse, Belgique) (Table 1).

**Table 1:** Classification and general features of *Clostridium ihumii* strain AP5^T^ according to the MIGS specification [18]

MIGS ID	Property	Term	Evidence code^a^
	Current classification	Domain Bacteria	TAS [19]
		Phylum *Firmicutes*	TAS [20–22]
		Class *Clostridia*	TAS [23, 24]
		Order *Clostridiales*	TAS [25, 26]
		Family *Clostridiaceae*	TAS [25, 27]
		Genus *Clostridium*	IDA [25, 28, 29]
		Species *Clostridium ihumii*	IDA
		Type strain AP5^T^	IDA
	Gram stain	Positive	IDA
	Cell shape	Rod-shapped	IDA
	Motility	Motile	IDA
	Sporulation	Sporulating	IDA
	Temperature range	Mesophile	IDA
	Optimum temperature	37 °C	IDA
	pH range; Optimum	Not determined	
	Carbon source	Not determined	
MIGS-6	Habitat	Human gut	IDA
MIGS-6.3	Salinity	Not determined	
MIGS-22	Oxygen requirement	Anaerobic	IDA
MIGS-15	Biotic relationship	free living	IDA
MIGS-14	Pathogenicity	unknown	
MIGS-4	Geographic location	France	IDA
MIGS-5	Sample collection time	January 2012	IDA
MIGS-4.1	Latitude	43.296482	IDA
MIGS-4.2	Longitude	5.36978	IDA
MIGS-4.3	Depth	Surface	IDA
MIGS-4.4	Altitude	0 m above sea level	IDA

^a^Evidence codes - IDA: Inferred from Direct Assay; TAS: Traceable Author Statement (i.e., a direct report exists in the literature); NAS: Non-traceable Author Statement (i.e., not directly observed for the living, isolated sample, but based on a generally accepted property for the species, or anecdotal evidence). These evidence codes are from the Gene Ontology project [30]

**Fig. 1:**
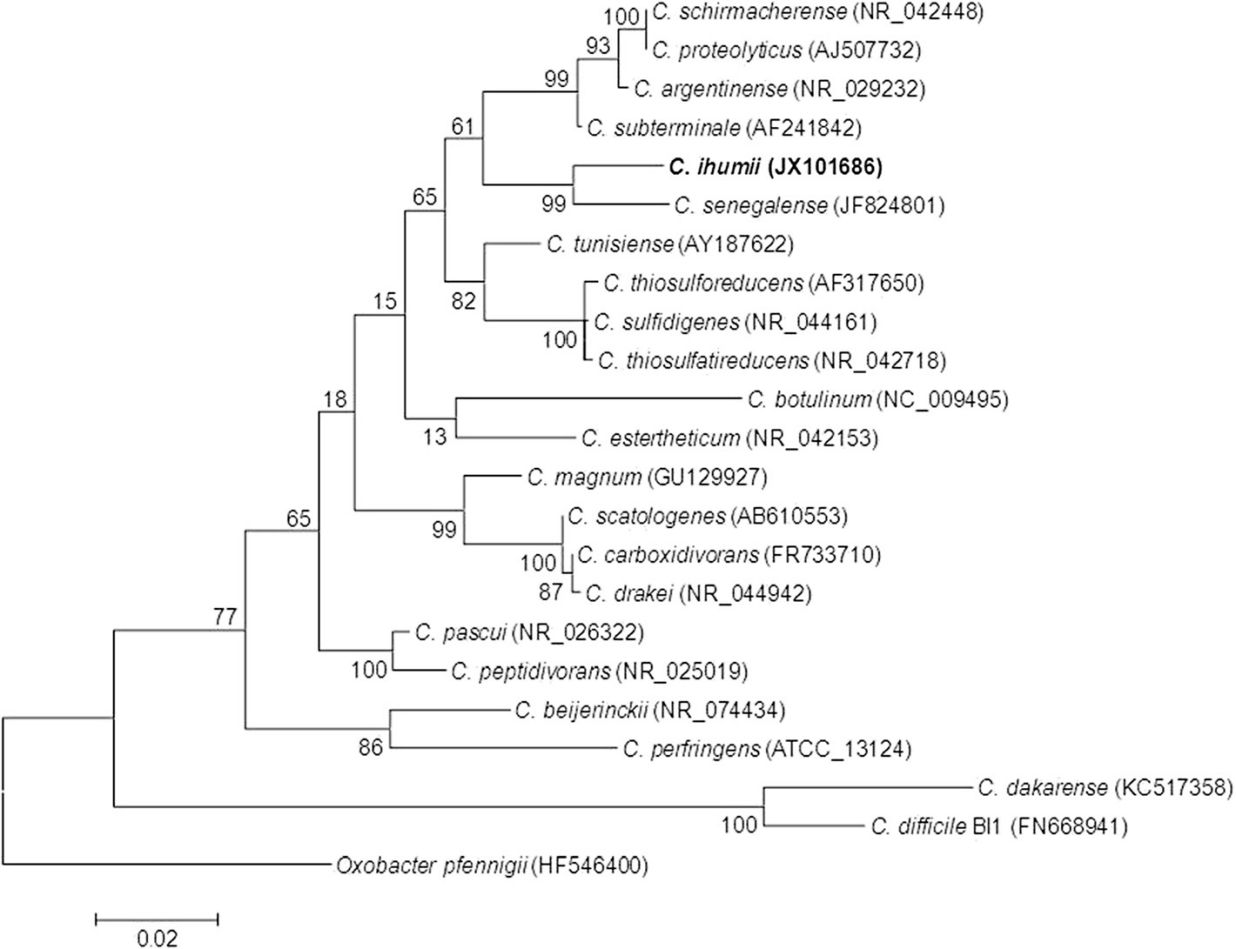
Phylogenetic tree highlighting the position of *C. ihumii* strain AP5^T^ relative to other type strains within the genus *Clostridium*. GenBank accession numbers are indicated in parentheses. Sequences were aligned using CLUSTALW, and phylogenetic inferences obtained using the maximum-likelihood method within the MEGA software. Numbers at the nodes are percentages of bootstrap values obtained by repeating 500 times the analysis to generate a majority consensus tree. *Oxobaxter pfennigii* was used as an outgroup. The scale bar represents 2 % nucleotide sequence divergence

The pairwise comparisons of the 16S rRNA sequence of *C. ihumii* strain AP5^T^ with that of the other validated *Clostridium* species yielded identity values ranging from 78.4 to 99.9 % in agreement with the values observed within the genus [31]. The highest value of nucleotide sequence similarity was observed with *Clostridium senegalense* (96.71 %), the phylogenetically closest species (Fig. 1). This value was lower than the 98.7 % 16S rRNA gene sequence threshold recommended by Stackebrandt and Ebers to delineate a new species without carrying out DNA-DNA hybridization [8].

**Fig. 2:**
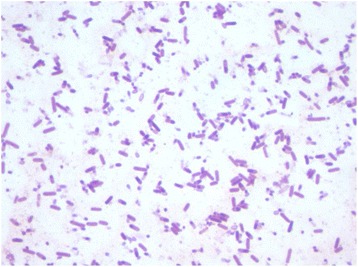
Gram staining of *C. ihumii* strain AP5^T^

**Fig. 3:**
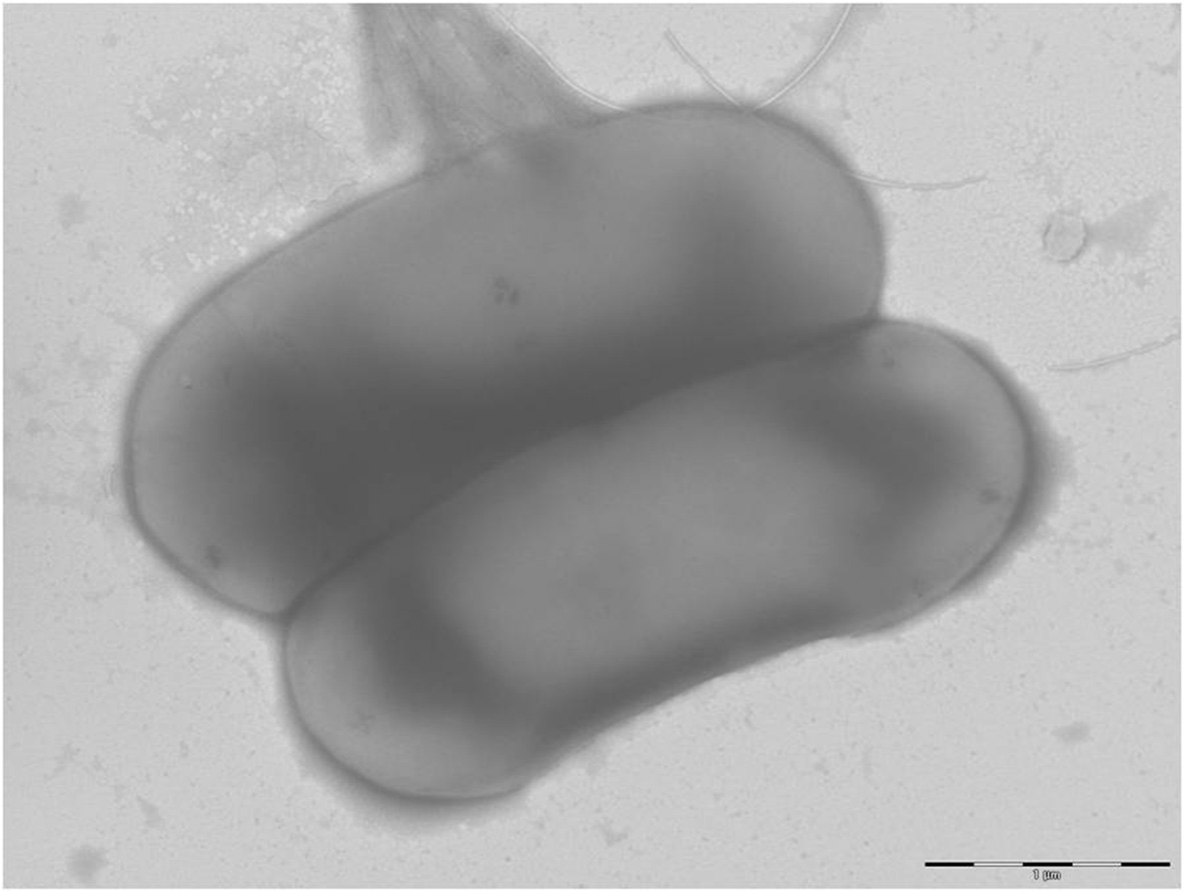
Transmission electron microscopy of *C. ihumii* strain AP5^T^ using a Morgani 268D (Philips) at an operating voltage of 60 kV. The scale bar represents 1um

The AP5^T^ strain was tested for growth on blood-enriched Columbia agar at different temperatures (25, 30, 37, 45 °C) and culture conditions (anaerobic and microaerophilic conditions using GENbag anaer and GENbag microaer systems, respectively (BioMerieux), and in aerobic conditions, with or without 5 % CO_2_ aerobic). Growth was observed only in anaerobic conditions and temperatures varying from 25 to 37 °C, with optimal growth at 37 °C. Colonies were 0.2-0.5 mm in diameter with smooth and white appearance. Gram staining showed Gram-positive rods able to form spores (Fig. 2). The motility test was positive. Cells grown on agar exhibit a mean diameter of 0.8 μm and a mean length of 1.5 μm as determined by negative staining transmission electron microscopy (Fig. 3).

The strain AP5^T^*C. ihumii* did neither have catalase nor oxidase activity (Additional file 1: Table S1). Using API 20 NE, API Rapid ID 32A strip and API ZYM (BioMerieux, Marcy l’Etoile), C. *ihumii* presented positive reactions for D-glucose and L-arabinose assimilation, arginine dihydrolase, esculin and gelatine hydrolysis, glutamic acid decarboxylase, alkaline and acid phosphatase, esterase, esterase lipase (C8), lipase (C14), α-galactosidase, β-galactosidase, β- glucuronidase, α-glucosidase, β-glucosidase, N-acetyl-β-glucosaminidase, alpha-mannosidase and arginine, proline, leucyl glycine, phenylalanine, leucine, pyroglutamic acid, tyrosine, alanine, glycine and histidine arylamidase. Negative reactions were observed for urease, nitrate reduction, indole production, D-mannose and D-maltose assimilation (Additional file 1: Table S1). *C. ihumii* is susceptible to amoxicillin, imipenem, metronidazole, rifampicin and vancomycin but resistant to trimethoprim/sulfamethoxazole.

**Fig. 4:**
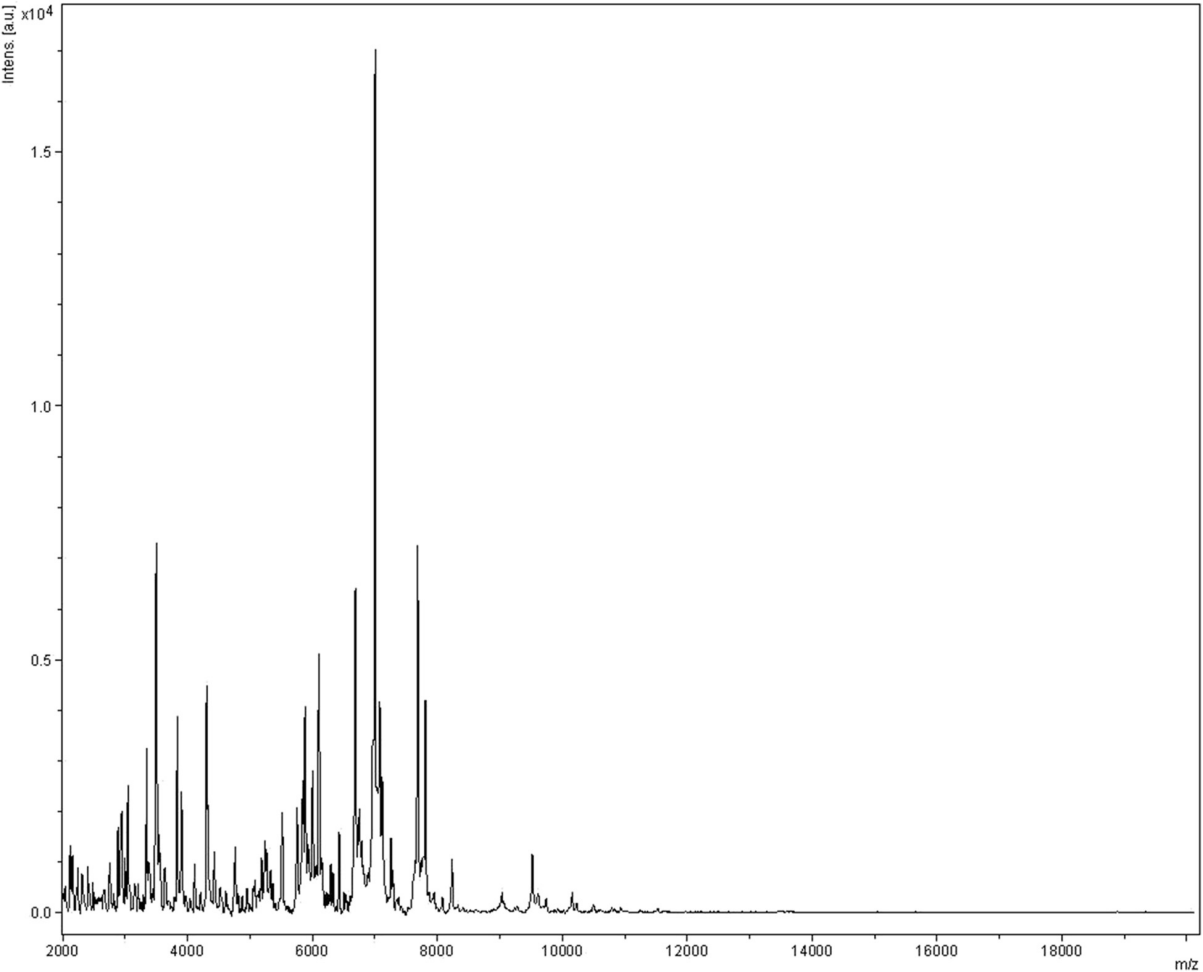
Reference mass spectrum from *C. ihumii* strain AP5^T^. Spectra from 12 individual colonies were compared and a reference spectrum was generated

**Fig. 5:**
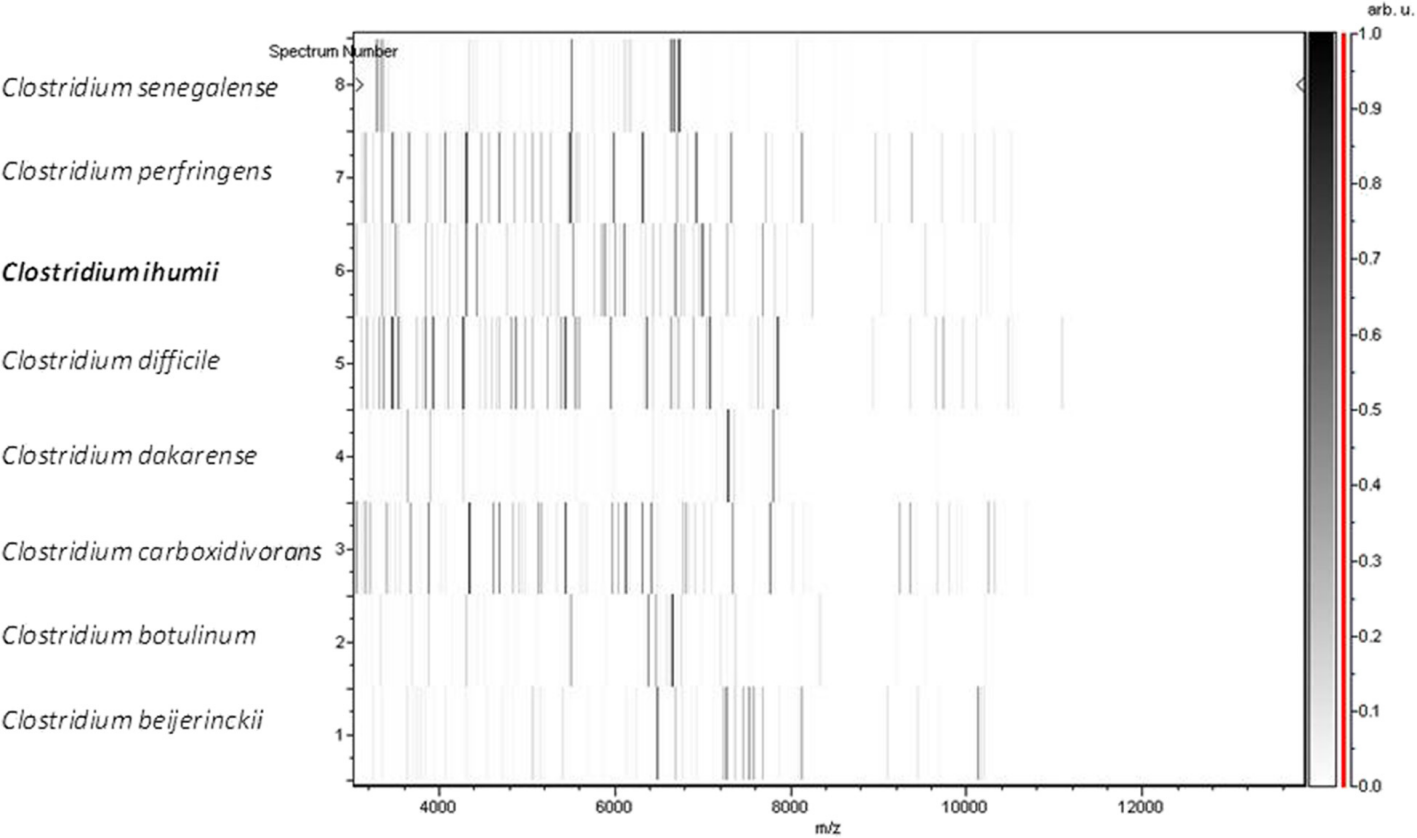
Gel view comparing *C. ihumii* strain AP5^T^ with *C. senegalense*, *C. perfringens*, *C. difficile*, *C. dakarense*, *C. carboxidivorans*, *C. botulinum* and *C. beijerinckii*, respectively. The gel view displays the raw spectra of loaded spectrum files arranged in a pseudo-gel like look. The x-axis records the m/z value. The left y-axis displays the running spectrum number originating from subsequent spectra loading. The peak intensity is expressed by a Gray scale scheme code. The color bar and the right y-axis indicate the relation between the color a peak is displayed with and the peak intensity in arbitrary units. The compared species are indicated on the left

Matrix-assisted laser-desorption/ionization time-of-flight (MALDI-TOF) MS protein analysis was carried out as previously described [32] using a Microflex spectrometer (Bruker Daltonics, Leipzig, Germany). Twelve isolated colonies of strain AP5^T^ were deposited on a MSP96 MALDI-TOF target plate. Each smear was overlaid with 2 μL of matrix solution (saturated solution of alpha-cyano-4-hydroxycinnamic acid) in 50 % acetonitrile, 2.5 % tri-fluoracetic acid, and allowed to dry for 5 minutes. Measurements were performed with a Microflex spectrometer (Bruker). Spectra were recorded in the positive linear mode for the mass range of 2,000 to 20,000 Da (parameter settings: ion source 1 (ISI), 20 kV; IS2, 18.5 kV; lens, 7 kV). A spectrum was obtained after 240 shots with variable laser power. The time of acquisition was between 30 seconds and 1 minute per spot. The twelve AP5^T^ spectra were imported into the MALDI BioTyper software (version 3.0, Bruker) and analyzed by standard pattern matching (with default parameter settings) against the main spectra of 7,316 bacteria, including 229 spectra from 97 *Clostridium* species, used as reference data, in the BioTyper database. The method of identification included the m/z from 3,000 to 15,000 Da. For every spectrum, a maximum of 100 peaks were compared with spectra in database. The resulting score enabled the identification of tested species, or not: score ≥ 1.9 with a validly published species enabled identification at the species level, a score ≥ 1.7 but < 1.9 enabled identification at the genus level, and a score < 1.7 did not enable any identification. No significant MALDI-TOF score was obtained for strain AP5^T^ against the Bruker database, suggesting that our isolate was not a member of a known species. We added the spectrum from strain AP5^T^ to our database (Fig. 4). The spectral differences with other members of the genus *Clostridium* are shown in the gel view (Fig. 5).

## Genome sequencing information

### Genome project history

The organism was selected for sequencing on the basis of its phylogenetic position and 16S rRNA similarity to members of the genus *Clostridium* and is part of a study of the human digestive flora aiming at isolating all bacterial species within human feces [1]. It was the 102nd genome from the genus *Clostridium* and the first genome of *C. ihumii* sp. nov. The EMBL accession number is CCAT000000000 and consists of 96 contigs. Table 2 shows the project information and its association with MIGS version 2.0 compliance [18].

**Table 2:** Project information

MIGS ID	Property	Term
MIGS-31	Finishing quality	High-quality draft
MIGS-28	Libraries used	Paired-end libraries
MIGS-29	Sequencing platforms	Roche 454 and MiSeq (Illumina)
MIGS-31.2	Fold coverage	32.7×
MIGS-30	Assemblers	CLC denovo assembly
MIGS-32	Gene calling method	Prodigal
	Locus Tag	BN346
	Genbank ID	CCAT000000000
	EMBL Date of release	03-20-2014
	BIOPROJECT	PRJEB373
MIGS-13	Source Material identifier	Human feces
	Project relevance	Study of the human gut microbiome

### Growth conditions and genomic DNA preparation

*C. ihumii* was grown on 5 % sheep blood-enriched Columbia agar (BioMerieux) at 37 °C in anaerobic atmosphere. Bacteria grown on three Petri dishes were harvested and resuspended in 4x100 μL of TE buffer. Then, 200 μL of this suspension was diluted in 1 ml TE buffer for lysis treatment. After a lysozyme incubation of 30 minutes at 37 °C the lysis was performed with lauryl sarcosyl by 1 % final and RNAseA treatment at 50μG/μL final concentration during 1 hr at 37 %°C followed by an overnight Proteinase K incubation at 37 °C. Extracted DNA was then purified using three successive phenol-chloroform extractions and ethanol precipitation at −20 °C overnight. After centrifugation, the DNA was resuspended in 70 μL TE buffer. The yield and concentration was measured by the Quant-it Picogreen kit (Invitrogen) on the Genios-Tecan fluorometer at 113 ng/μl.

### Genome sequencing and assembly

The genome was sequenced using two high throughput NGS technologies: Roche 454, and MiSeq Technology (Illumina Inc, San Diego, CA, USA) with the paired end application. For the construction of 454 library, 5 μg of DNA was mechanically fragmented on the Covaris device (KBioScience-LGC Genomics, Queens Road, Teddington, Middlesex, TW11 0LY, UK) through miniTUBE-Red 5Kb. The DNA fragmentation was visualized through the Agilent 2100 BioAnalyzer on a DNA labchip 7500 with an optimal size of 3.9 kb, an insert size smaller than expected. Circularization and fragmentation were performed on 100 ng. The 454 library was loaded on a quarter region of the GS Titanium PicoTiterPlate PTP and sequenced with the GS FLX Titanium Sequencer (Roche). After PCR amplification through 17 cycles followed by double size selection, the single stranded paired end library was then loaded on a DNA labchip RNA pico 6000 on the BioAnalyzer: The pattern showed an optimal at 545 bp. The library was then quantified on the Quant-it Ribogreen kit (Invitrogen) using a Genios Tecan fluorometer at 10,190 pg/μL. The library concentration equivalence was calculated as 2.37E + 10 molecules/μL. The library was clonally amplified at 0.5 and 1 cpb in 2 emPCR reactions per conditions with the GS Titanium SV emPCR Kit (Lib-L) v2 (Roche). The yield of the emPCR was 5.2 and 12.5 % according to the quality expected by the range of 5 to 20 % from the Roche procedure, respectively. These two enriched clonal amplifications were loaded with 790,000 beads on the GS Titanium PicoTiterPlates PTP Kit 70x75 and sequenced with the GS Titanium Sequencing Kit XLR70. The run was performed in overnight and then analyzed on the cluster through the gsRunBrowser and gsAssembler_Roche. A total of 246,499 filter-passed wells were obtained. They generated 98.64 Mb of DNA sequences with a length average of 400 bp.

The library for Illumina was prepared using the Nextera XT technology. The DNAg was quantified by a Qubit assay with the high sensitivity kit (Life technologies, Carlsbad, CA, USA) and diluted to require 1 ng of genome as input to prepare the paired end library. DNA was fragmented and tagged during the “tagmentation” step with an optimal size distribution at 0.85 kb. Limited cycle PCR amplification (12 cycles) completed the tag adapters and introduced dual-index barcodes. After purification on AMPure XP beads (Beckman Coulter Inc, Fullerton, CA, USA), the library was normalized and loaded onto the reagent cartridge and then onto the instrument along with the flow cell. Automated cluster generation and paired-end sequencing with dual index reads were performed in single 39-hours run in 2x250-bp. Total information of 6.83 Gb was obtained from a 807 K/mm2 cluster density with a cluster passing quality control filters of 90.88 % (14,553,000 clusters); 3.14 % of this total information concerned the sequencing of *Clostridium**ihumii* (415,280 passed filter clusters). Sequences obtained with Roche were assembled on the gsAssembler with 90 % identity and 40 bp of overlap. It leads to 397 large contigs (>1500 bp) arranged into 21 scaffolds and generated a genome size of 1.62 Mb which corresponds to a coverage of 60.88× genome equivalent. Sequencing through Illumina MiSeq resulted in 415,280 reads that assembled in 1077 contigs. Both platform data was used for the hybrid assembly. A total of 1,143,611 high-quality reads with approximately 32.7× coverage were assembled using CLC Genomics Workbench v. 6.0 (CLC bio, Katrinebjerg, Denmark) generating 96 large contigs with total length of 4.43 Mbp.

### Genome annotation

Open Reading Frames were predicted using Prodigal [33] with default parameters. However, the predicted ORFs were excluded if they spanned a sequencing gap region. The predicted bacterial protein sequences were searched against the GenBank [34] and Clusters of Orthologous Groups databases using BLASTP. The tRNAs and rRNAs were predicted using the tRNAScanSE [35] and RNAmmer [36] tools, respectively. Lipoprotein signal peptides and numbers of transmembrane helices were predicted using SignalP [37] and TMHMM [38], respectively. ORFans were identified if their BLASTP *E*-value was lower than 1e^−03^ for alignment length greater than 80 amino acids. If alignment lengths were smaller than 80 amino acids, we use an *E*-value of 1e^−05^. Such parameter thresholds have already been used in previous works to define ORFans.

Artemis [39] and DNA Plotter [40] were used for data management and visualization of genomic features, respectively. Mauve alignment tool (version 2.3.1) was used for multiple genomic sequence alignment [41]. To estimate Average Genome Identity of Orthologous Sequences [10] at the genome level between *C. ihumii* and another 7 members of the *Clostridium* genus, orthologous proteins were detected using the Proteinortho [42] and we compared genomes two by two and determined the mean percentage of nucleotide sequence identity among orthologous ORFs using BLASTn.

## Genome properties

**Fig. 6:**
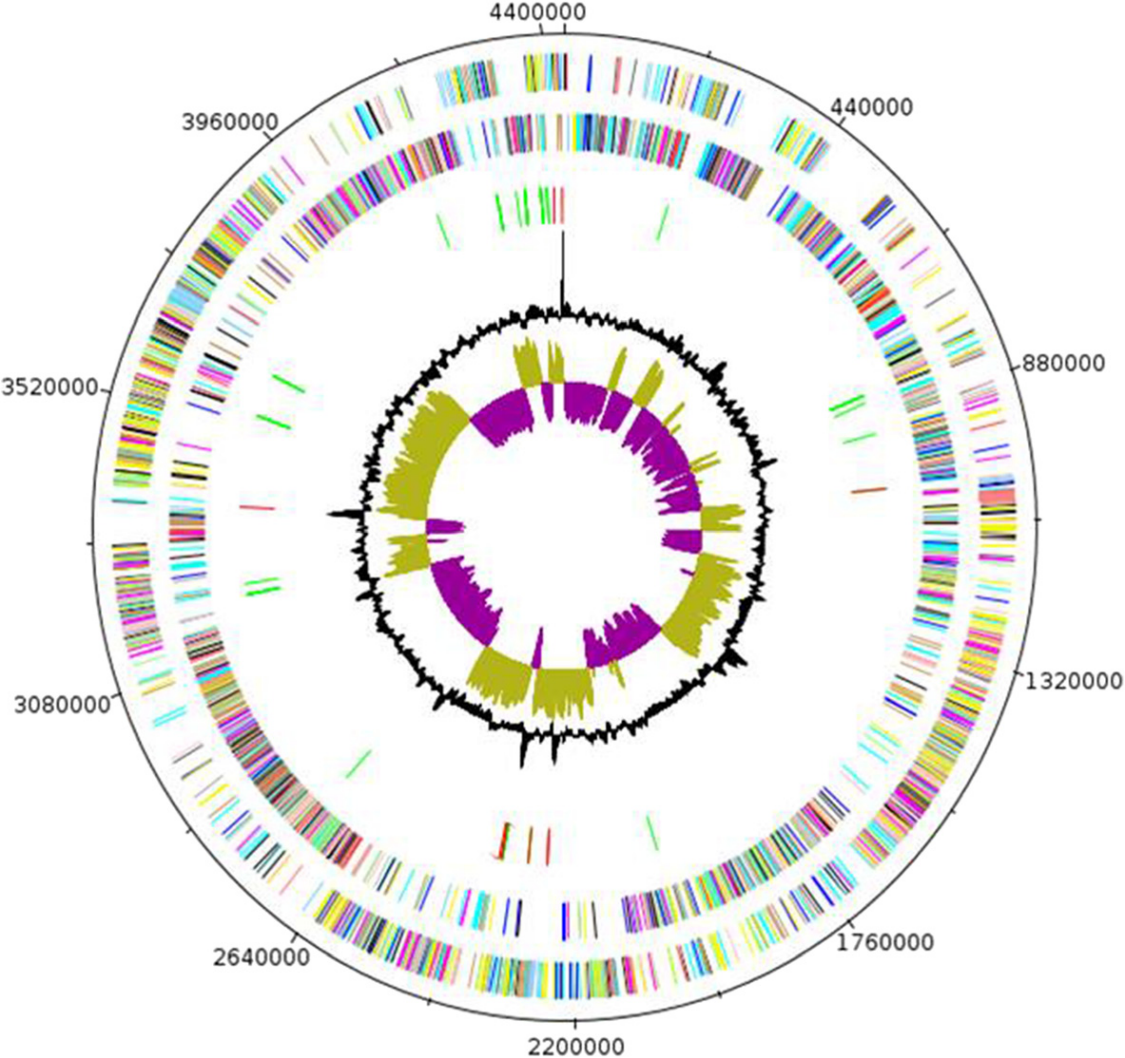
Graphical circular map of the chromosome. From the outside in, the outer two circles show open reading frames oriented in the forward and reverse directions (colored by COG categories), respectively. The third circle shows the rRNA gene operon (red) and tRNA genes (green). The fourth circle shows the G + C% content plot. The inner-most circle shows GC skew, purple and olive indicating negative and positive values, respectively

The genome is 4,433,668 bp long (one chromosome, no plasmid) with a GC content of 26.70 % (Fig. 6 and Table 3). Of the 4,161 predicted chromosomal genes, 4,076 were protein-coding genes and 85 were RNAs including 79 tRNAs and 9 rRNAs (5S = 4, 23S = 3, 16S = 2). A total of 2,408 genes (57.83 %) were assigned a putative function. Two hundred and ninety two genes were identified as ORFans (7.01 %) and the remaining genes were annotated as hypothetical proteins. The properties and statistics of the genome are summarized in Tables 2 and 3. The distribution of genes into COGs functional categories is presented in Table 4.

**Table 3:** Genome statistics

Attribute	Value	% of Total^a^
Genome size (bp)	4,433,668	100
DNA coding (bp)	3,646,866	82.25
DNA G + C (bp)	1,183,789	26.70
DNA scaffolds	21	
Total genes	4,161	100
Protein coding genes	4,076	97.96
RNA genes	85	2.04
Pseudo genes	ND	
Genes in internal clusters	ND	
Genes with function prediction	2,408	57.83
Genes assigned to COGs	2,777	66.74
Genes with Pfam domains	ND	
Genes with signal peptides	84	2.02
Genes with transmembrane helices	1,107	26.60
CRISPR repeats	ND	

^a^ The total is based on either the size of the genome in base pairs or the total number of protein coding genes in the annotated genome. ND for Not determined

**Table 4:** Number of genes associated with general COG functional categories

Code	Value	% of total^a^	Description
J	208	4.60	Translation
K	292	6.50	Transcription
L	181	4.02	Replication, recombination and repair
D	31	0.69	Cell cycle control, mitosis and meiosis
V	158	3.51	Defense mechanisms
T	226	5.02	Signal transduction mechanisms
M	154	3.42	Cell wall/membrane biogenesis
N	92	2.04	Cell motility
U	43	0.95	Intracellular trafficking and secretion
O	86	1.91	Posttranslational modification, protein turnover, chaperones
C	165	3.66	Energy production and conversion
G	101	2.24	Carbohydrate transport and metabolism
E	229	5.09	Amino acid transport and metabolism
F	78	1.73	Nucleotide transport and metabolism
H	119	2.64	Coenzyme transport and metabolism
I	66	1.47	Lipid transport and metabolism
P	163	3.62	Inorganic ion transport and metabolism
Q	72	1.60	Secondary metabolites biosynthesis, transport and catabolism
R	493	10.95	General function prediction only
S	245	5.44	Function unknown
-	1299	28.85	Not in COGs

^a^ The total is based on the total number of protein coding genes in the annotated genome

## Insights from the genome sequence

**Fig. 7:**
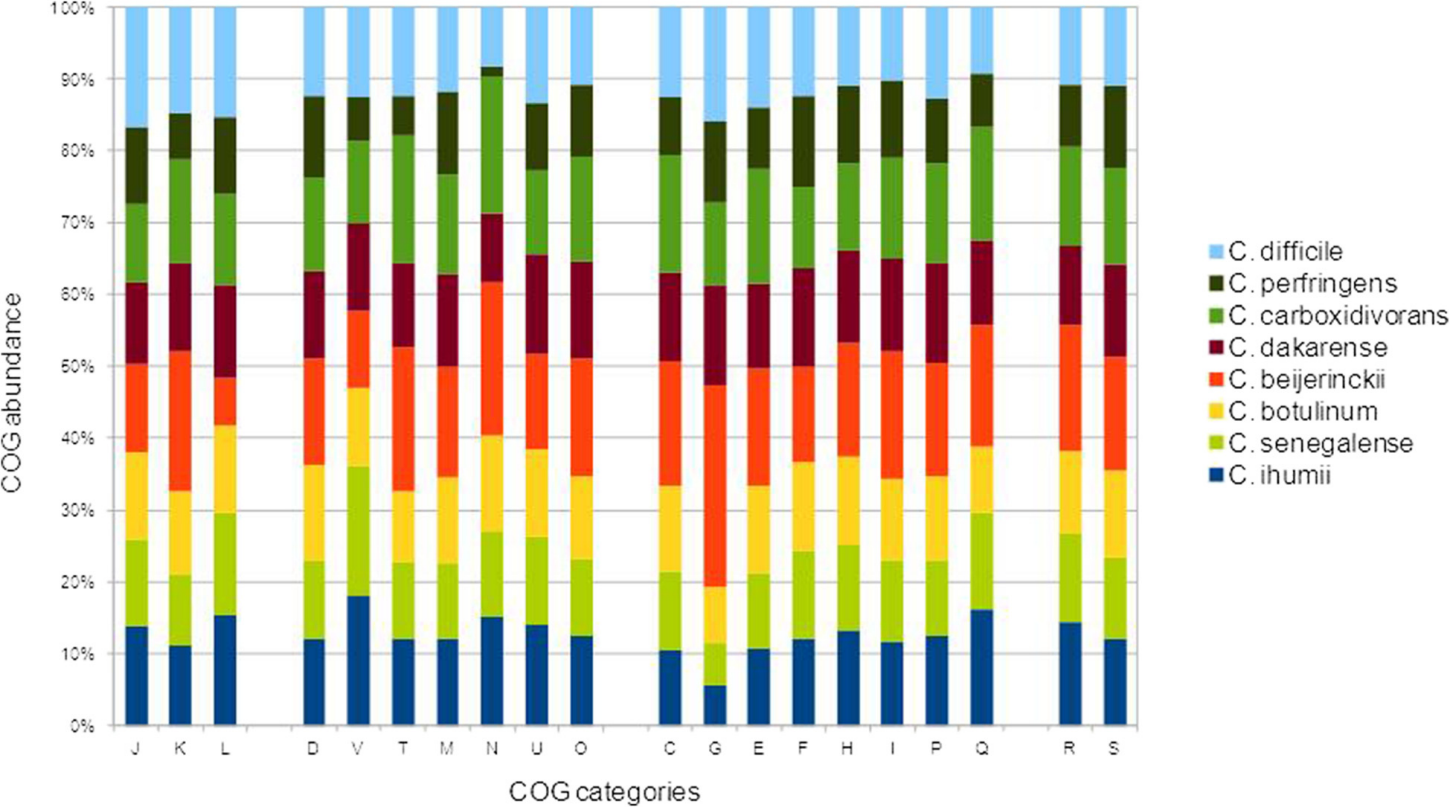
Distribution of functional classes of predicted genes in the *C. ihumii* AP5^T^ and other 7 *clostridium* genomes according to the clusters of orthologous groups of proteins

We compared the genome of *C. ihumii* strain AP5^T^ with those of *C. beijerinckii* strain NCIMB 8052, *C. botulinum* strain ATCC 3502, C. *carboxidivorans* strain P7, *C. dakarense* strain FF1, *C. difficile* strain B1, *C. perfringens* strain AGR 2156 and *C. senegalense* strain JC122 (Table 5A and B). The draft genome sequence of *C.* ihumii strain AP5^T^ is smaller than those of *C. beijerinckii**and**C. difficile* (6.0 and 4.46 Mb, respectively), but larger than those of *C. carboxidivorans*, *C. botulinum*, *C. senegalense**,**C. dakarense**,**C. perfringens*, and (4.41, 3.9, 3.89, 3.73 and 3.26 Mb, respectively). The G + C content of *C. ihumii* is the lowest among the compared genomes. The gene content of *C.ihumii* is smaller than those of *C. beijerinckii* and *C. carboxidivorans*, (5,020 and 4,174, respectively) but larger those of *C. dakarense*, *C. senegalense**,**C. difficile*, *C. botulinum* and *C. perfringens* and (3,818, 3,704, 3,591, 3,572 and 2,876, respectively). The distribution of genes into COG categories was not entirely similar in all the 8 compared genomes (Fig. 7).

**Table 5:** Genomic comparison of *C. ihumii* with 7 other members of *Clostridium* species

A								
Species			Strain		Genome accession number		Genome size (Mb)	G + C content
*C. ihumii*			AP5		CCAT000000000		4.43	26.7
*C. perfringens*			ATCC 13124		NC_008261		3.26	28.4
*C. dakarense*			FF1		CBTZ010000000		3.73	27.9
*C. senegalense*			JC122		CAEV01000001		3.89	26.8
*C. botulinum*			ATCC 3502		NC_009495		3.90	28.2
*C. carboxidivorans*			P7		NZ_ADEK00000000		4.41	29.7
*C. difficile*			B1		NC_017179		4.46	28.4
*C. beijerinckii*			NCIMB 8052		NC_009617		6.00	29.0
B								
	*C. ihu*	*C. per*	*C. dak*	*C. sen*	*C. bot*	*C. car*	*C. dif*	*C. bej*
*C. ihu*	4,076	1185	1189	1688	1427	1186	1124	1310
*C. per*	72.10	2,876	1080	1173	1132	978	996	1268
*C. dak*	70.40	70.36	3,818	1156	1099	1022	1307	1189
*C. sen*	79.10	72.14	70.34	3,704	1442	1183	1095	1292
*C. bot*	72.58	72.01	69.74	73.10	3,572	1342	1143	1450
*C. car*	72.17	71.57	69.40	72.48	74.09	4,174	1046	1342
*C. dif*	69.70	69.55	77.68	69.53	69.18	69.08	3,591	1194
*C. bej*	70.85	71.97	69.10	71.10	71.01	71.45	68.52	5,020

*C.ihu* = *C. ihumii*, *C. bej* = *C. beijerinckii*, *C. bot* = *C. botulinum*, *C. car* = *C. carboxidivorans*, *C. dak* = *C. dakarense*, *C. dif* = *C. difficile*, *C. per* = *C. perfringens*, *C. sen* = *C. senegalense*

**a**: Species name, Strain, EMBL and GenBank accession number, Genome size and GC content of compared genomes

**b**: Numbers of orthologous protein shared between genomes (upper right triangle), average percentage similarity of nucleotides corresponding to orthologous proteins shared between genomes (lower left triangle) and the numbers of proteins per genome (bold diagonal)

In addition, *C.* ihumii shared 1,688, 1,427, 1,310, 1,189, 1,186, 1,185 and 1,124 genes with those of *C. senegalense*, *C. botulinum*, *C. beijerinckii**,**C. dakarense*, *C. carboxidivorans*, *C. perfringen.* and *C. difficile*, respectively. Among compared genomes except for *C. ihumii*, AGIOS values ranged from 68.52 between *C. difficile* and *C. beijerinckii* to 77.68 % between *C. difficile* and *C. dakarense*. When *C.* ihumii was compared to other species*,* AGIOS values ranged from 70.85 with *C. beijerinckii* to 79.10% with *C. senegalense* (Table 5B).

## Conclusion

On the basis of phenotypic, phylogenetic and genomic analyses, we formally propose the creation of *Clostridium**ihumii* sp. nov. that contains strain AP5^T^. This bacterial strain was isolated from the fecal flora of an anorexia nervosa woman living in Marseille, France.

### Description of *Clostridium* ihumii sp. nov.

*Clostridium**ihumii* (i.hum.i’i. N.L. gen. n. ihumii, based on the acronym IHUMI, the Institut Hospitalo-Universitaire Méditerranée-Infection in Marseille, France, where the type strain was isolated). The type strain AP5^T^ (=CSUR P198 = DSM 26098) was obtained from the fecal flora of a patient with anorexia. Growth was observed at different temperatures between 25 and 37 °C on axenic medium in strict anaerobic conditions. Colonies were smooth and white with 0.2-0.5 mm in diameter, on blood-enriched Columbia agar. Cells stain Gram-positive, they are rod-shaped, endospore-forming, non-motile and have a mean diameter of 0.8 μm and a mean length of 1.5 μm.

Catalase, oxidase, urease and indole production are absent. Arginine dihydrolase, α-glucosidase, β-glucosidase, N-acetyl-β-glucosaminidase are present. Cells are sensitive to amoxicillin, imipenem, metronidazole, rifampicin and vancomycin but resistant to trimethoprim/sulfamethoxazole.

The G + C content of the genome is 26.7 %. The 16S rRNA and genome sequences were deposited in GenBank and EMBL under accession numbers JX101686 and CCAT000000000, respectively.

## Additional file

Additional file 1: Table S1. Differential characteristics of *C.* ihumii AP5^T^, *Clostridium* beijerinckii strain NCIMB 8052, C*lostridium botulinum* strain ATCC 3502, C*lostridium carboxidivorans* strain P7, C*lostridium* dakarensestrain FF1, *Clostridium difficile* strain B1, C*lostridium* perfringens strain ATCC 13124, and *C. senegalense* strain JC122.

## Abbreviations

AGIOS: Average Genome Identity of Orthologous Sequences
