# Supplementary material for: Non-contiguous finished genome sequence and description of Clostridiumihumii sp. nov
Source: Stand Genomic Sci. 2015 Sep 19;10:63. doi: 10.1186/s40793-015-0025-x (PMC4575456; doi:10.1186/s40793-015-0025-x)
Supplement: Additional file 1: Table S1. — Differential characteristics of C. ihumii AP5T, Clostridium beijerinckii strain NCIMB 8052, Clostridium botulinum strain ATCC 3502, Clostridium carboxidivorans strain P7, Clostridium dakarensestrain FF1, Clostridium difficile strain B1, Clostridium perfringens strain ATCC 13124, and C. senegalense strain JC122. [file 40793_2015_25_MOESM1_ESM.docx]

**Additional file 1**

**Table S1:** Differential characteristics of [*C.* ihumii](http://dx.doi.org/10.1601/nm.25954) AP5^T^, [*Clostridium* beijerinckii](http://dx.doi.org/10.1601/nm.3898) strain [NCIMB 8052](http://doi.org/10.1601/strainfinder?urlappend=%3Fid%3DNCIMB+8052), [C*lostridium botulinum*](http://dx.doi.org/10.1601/nm.3901) strain [ATCC 3502](http://doi.org/10.1601/strainfinder?urlappend=%3Fid%3DATCC+3502), [C*lostridium carboxidivorans*](http://dx.doi.org/10.1601/nm.9596) strain P7, [C*lostridium*](http://dx.doi.org/10.1601/nm.3878) dakarensestrain FF1, [*Clostridium difficile*](http://dx.doi.org/10.1601/nm.3924) strain B1, [C*lostridium* perfringens](http://dx.doi.org/10.1601/nm.3991) strain [ATCC 13124](http://doi.org/10.1601/strainfinder?urlappend=%3Fid%3DATCC+13124), and [*C. senegalense*](http://dx.doi.org/10.1601/nm.23563) strain JC122.

| **Properties** | [***C. ihumii***](http://dx.doi.org/10.1601/nm.25954) | [***C. senegalense***](http://dx.doi.org/10.1601/nm.23563) | [***C. beijerinckii***](http://dx.doi.org/10.1601/nm.3898) | [***C. botulinum***](http://dx.doi.org/10.1601/nm.3901) | [***C. dakarense***](http://dx.doi.org/10.1601/nm.25962) | [***C. difficile***](http://dx.doi.org/10.1601/nm.3924) | [***C. perfringens***](http://dx.doi.org/10.1601/nm.3991) |
| --- | --- | --- | --- | --- | --- | --- | --- |
| Cell diameter (µm) | 1.5 | 3.0 | 1.7 | 1.5 | 1.5 | 1.1 | 1.2 |
| Oxygen requirement | Strictly  anaerobic | Strictly  anaerobic | Strictly  anaerobic | Strictly  anaerobic | Strictly  anaerobic | Strictly  anaerobic | Strictly  anaerobic |
| Gram stain | Positive | Variable | Variable | Positive | Positive | Positive | Positive |
| Motility | Motile | Motile | Motile | Na | Motile | Motile | Motile |
| Endospore formation | + | + | + | Na | + | + | + |
| Catalase | - | Na | - | - | - | - | - |
| Oxidase | - | Na | Na | Na | - | - | - |
| Urease | - | Na | - | Na | - | - | - |
| Indole production | - | Na | Na | - | - | - | + |
| Nitrate reductase | + | - | - | Na | - | - | - |
| Alkaline phosphatase | + | Na | Na | Na | Na | - | + |
| β-galactosidase | - | Na | Na | Na | Na | - | - |
| N-acetyl-β-glucosaminidase | - | Na | Na | Na | Na | + |  |
| **Assimilation** |  |  |  |  |  |  |  |
| D-glucose | + | Na | + | + | + | Na | + |
| L-Arabinose | + | - | + | Na | + | Na | - |
| D-mannose | - | + | + | + | + | Na | - |
| D-mannitol | - | + | + | + | + | Na | - |
| D-maltose | - | - | + | + | + | Na | + |
| **G+C content (%)** | 27 | 28 | 28 | 29 | 31 | 27 | 28 |
| **Habitat** | Human gut | Human gut | Human gut | Rat gut | Environment | Human gut | Human gut |

Na = data not available
